# Acute Kidney Injury in Neonatal Intensive Care Unit: Epidemiology, Diagnosis and Risk Factors

**DOI:** 10.3390/jcm13123446

**Published:** 2024-06-13

**Authors:** Valeria Chirico, Antonio Lacquaniti, Filippo Tripodi, Giovanni Conti, Lucia Marseglia, Paolo Monardo, Eloisa Gitto, Roberto Chimenz

**Affiliations:** 1 Pediatric Nephrology and Dialysis Unit, University Hospital “G. Martino”, 98124 Messina, Italytripodifilippo1@gmail.com (F.T.);; 2 Nephrology and Dialysis Unit, Papardo Hospital, 98158 Messina, Italypmonardo66@gmail.com (P.M.); 3 Neonatal and Pediatric Intensive Care Unit, Department of Human Pathology in Adult and Developmental Age “Gaetano Barresi”, University of Messina, 98124 Messina, Italy; luciamarina.marseglia@unime.it (L.M.);

**Keywords:** acute kidney injury, neonatal intensive care unit, nephrotoxic drugs, renal biomarkers, renal injury, sepsis

## Abstract

Acute kidney injury (AKI) is associated with long-term consequences and poor outcomes in the neonatal intensive care unit. Its precocious diagnosis represents one of the hardest challenges in clinical practice due to the lack of sensitive and specific biomarkers. Currently, neonatal AKI is defined with urinary markers and serum creatinine (sCr), with limitations in early detection and individual treatment. Biomarkers and risk factor scores were studied to predict neonatal AKI, to early identify the stage of injury and not the damage and to anticipate late increases in sCr levels, which occurred when the renal function already began to decline. Sepsis is the leading cause of AKI, and sepsis-related AKI is one of the main causes of high mortality. Moreover, preterm neonates, as well as patients with post-neonatal asphyxia or after cardiac surgery, are at a high risk for AKI. Critical patients are frequently exposed to nephrotoxic medications, representing a potentially preventable cause of AKI. This review highlights the definition of neonatal AKI, its diagnosis and new biomarkers available in clinical practice and in the near future. We analyze the risk factors involving patients with AKI, their outcomes and the risk for the transition from acute damage to chronic kidney disease.

## 1. Introduction

Acute kidney injury (AKI), frequently involving patients admitted to the neonatal intensive care unit (NICU), is associated with poor outcomes and affects 18–70% of critically ill neonates [1,2]. Although the real incidence of AKI in neonates is uncertain, with wide and various ranges, due to several definitions and diagnostic methods, more immature and ill neonates are characterized by the highest risk of AKI [3]. Patients with congenital heart disease, perinatal asphyxia, premature birth or a low birth weight, and necrotizing enterocolitis, as well as neonates who receive nephrotoxic medications or require extracorporeal life support, represent high-risk populations.

According to the Worldwide Acute Kidney Injury Epidemiology in Neonates (AWAKEN) data, one episode of AKI, related to an increased length of hospitalization and mortality rate, was observed in 30% of critically ill neonates [4].

To apply personalized therapeutic approaches to AKI, an adequate and early diagnosis could reduce or avoid subclinical and precocious renal injuries and their related effects, requiring chronic follow-up, as suggested by the Clinical Practice Guidelines for Acute Kidney Injury (KDIGO), recommending a nephrological evaluation after three months, if an AKI event occurs [5,6]. The precocious diagnosis of AKI based on urinary output and serum creatinine (sCr) levels represents one of the hardest challenges in clinical practice. Several biomarkers and clinical scores were assessed to predict neonatal AKI, to identify the stage of injury and not the damage, to anticipate late rises in sCr values and to reveal an already compromised renal function.

This review highlights the definition of neonatal AKI, its diagnosis and new biomarkers available in clinical practice and the near future. We analyze the risk factors of patients with AKI, their outcomes and the risk of the transition from acute damage to chronic kidney disease (CKD).

## 2. Diagnosis of AKI: State of the Art and Future Perspectives

### 2.1. Neonatal Creatinine-Based AKI Definitions

The KDIGO stages, applied in adults, are commonly adopted in children but without guidelines for pediatric use.

Assuming that a definition should evaluate the actual incidence of a pathological condition and its related events, pediatric risk, injury, failure, loss, end-stage renal disease (pRIFLE), Acute Kidney Injury Network (AKIN) and KDIGO criteria were compared in children to establish their reliability in evaluating AKI. In this context, the KDIGO definition was considered the most adequate for the pediatric population, highlighting the need for a unified AKI definition [7]. However, the diagnosis of AKI in neonates is difficult for several reasons. Neonates exhibit non-oliguric kidney failure, which is often related to toxin exposition [8]. Unfortunately, sCr is an unreliable indicator in the early phases of injury, modified by confounding factors such as race, muscle mass, sepsis and rhabdomyolysis [9]. As in adulthood, sCr levels remain within the normal range even if non-negligible kidney function of about 25 to 50% is lost, not revealing the initial decline in the renal reserve [10]. If an acute decline in glomerular filtration occurs, sCr does not reveal early kidney dysfunction, increasing only 2–3 days after injury [11].

The immaturity of the kidneys during the pediatric age represents another limitation of not having standardized definitions for detecting AKI, with more difficulties in premature or seriously ill babies. sCr changes during development and its relationship with age and baseline levels have not been established in children. Concentrations of sCr depend on the growth of children, indicating that kidney function limits must be determined according to gestational age and developmental periods. The CALIPER study demonstrated different concentrations of creatinine during child growth and development, suggesting the need for a continuous reference interval reflecting this dynamic change, rather than static age- and sex-related reference values [12].

Starting from these assumptions, a large multicenter study in children evaluated if sCr changes, according to age and the initial creatinine value, indicate an AKI event, comparing the data with KDIGO and pRIFLE criteria, based on a fixed percentage increase in creatinine. This study revealed that the dynamic reference change value of creatinine according to age, established by a pediatric reference change value optimized for AKI in children (pROCK), estimated that greater than 20 μmol/L or 30% of the initial creatinine level improves the detection of “true” AKI compared with earlier definitions that may lead to pediatric AKI overdiagnosis [13].

Furthermore, the pROCK criteria have been applied to critically ill children for AKI detection, revealing that, at ICU admission, this method better correlated with the severity and outcome of AKI if compared to pRIFLE and KDIGO definitions [14].

Contrasting data have emerged in the literature data, indicating that the pROCK criteria exclude a large proportion of children with AKI stage 1 defined by KDIGO, avoiding overdiagnosis but, at the same time, not detecting the initial loss of the renal reserve and not allowing clinicians to pursue precocious and preventive nephroprotective strategies [15,16].

Furthermore, hyperglycemia, high total protein and hemolysis are responsible for false elevations in sCr [17].

Published data on preterm infants revealed that high sCr levels are observed in the first 48 h of life, especially in infants of <30 weeks of gestation, leading to the hypothesis that reduced clearance of a newborn’s kidney can eliminate maternal creatinine loads [18]. Moreover, increased sCr levels in newborns could be due to the reduced clearance caused by a reduced glomerular filtration rate (GFR) and tubular reabsorption of creatinine [19]. For these reasons, creatinine cannot be considered an adequate marker for the diagnosis of acute kidney neonatal injury.

AKI management in the NICU is related to the fluid management of patients and the assessment of water balance. Fluid overload and AKI are cause-and-effect potentiators, with a high risk of detecting falsely reduced creatinine, underestimating AKI [20].

In intensive care, fluid management is often based on massive volume expansion, especially following surgery or resuscitation for sepsis, increasing the total body water by more than 10% within 72 h, reducing sCr concentrations and underestimating the severity of kidney injury [21].

Renal replacement therapy (RRT) “timing” is based on the severity of fluid overload, as well as severe acidosis or electrolyte imbalance, i.e., refractory hyperkalemia, behind a single value of sCr. All confounding risk factors to renal failure determined individually are analyzed, and the treatment is personalized [22].

In this context, peritoneal dialysis (PD) is preferred in neonates with AKI, whereas major abdominal surgery, severe hyperammonaemia, necrotizing enterocolitis and abdominal wall defects represent the main contraindications. The risk of leaking is higher, and thereafter, so is infection after catheter placement [23].

### 2.2. New Biomarkers of AKI

In the past decades, growing literature has focused on renal biomarkers to assess AKI events in early stages, improve clinical decisions and allow for personalized treatment [24]. Several clinical randomized trials have evaluated the sensitivity and specificity of glomerular and tubular biomarkers, including neutrophil gelatinase-associated lipocalin (NGAL) [25], interleukin (IL)-18 [26], kidney injury molecule-1 (KIM-1) [27,28], β-2 microglobulin (β-2MG) and Cystatin-C [29].

Newborns undergoing cardiopulmonary bypass were the most studied population, but further research should evaluate these biomarkers in other critical pediatric populations, such as very-low-birth-weight (VLBW) neonates [30].

For the same biomarker, urine evaluation can provide more specific information about renal injury than serum analysis, avoiding the influence of systemic inflammation or alterations due to concomitant non-renal diseases [31].

However, more works are needed to validate all these following urinary and sera biomarkers, which are not used in common clinical practice, with the exception of cystatin C.

#### 2.2.1. Neutrophil Gelatinase-Associated Lipocalin (NGAL)

Neutrophil gelatinase-associated lipocalin (NGAL) is a component of innate immunity, released by activated neutrophils and epithelial cells of several tissues, including the renal thick limbs of Henle and collecting ducts [32]. NGAL, detectable in the blood and urine precociously, represents a potential AKI biomarker, highlighting hypoxic–ischemic damage involving renal tubular epithelial cells [33].

In particular, high urine and serum NGAL levels early revealed AKI after cardiac surgery [30]. In a prospective trial that enrolled critically ill children, urine NGAL predicted AKI before an increase in SCr [34,35,36], as well as in premature infants with AKI, in whom high urinary NGAL concentrations were independently associated with mortality [37,38]. Similarly, Merve showed higher NGAL concentrations in 70 premature calves with respiratory distress syndrome and AKI, closely related to a 4-fold high mortality risk [39].

Another clinical application of this biomarker is the detection of hypoxic–ischemic AKI, responsible for renal tubular epithelial cell damage, with increased urine and serum NGAL concentrations [33]. Moreover, NGAL is an early predictive biomarker of contrast-induced nephropathy in children [40].

In particular, nephrotoxic medication exposure in NICU induces an increase in urine NGAL, with a risk for AKI of 2.76 for urine NGAL levels ≥ 400 ng/mL [41].

A prospective observational study involving very low birth weight assessed the reference range of urine NGAL concentrations. The median, the 25th, 75th and 95th quantiles and the range of pooled urine NGAL concentrations were 5 ng/mL, 2 ng/mL, 10 ng/mL, 50 ng/mL and 2–150 ng/mL, respectively. These data are not valid in patients with gestations < 26 weeks or with birth weights ≤ 750 g with uncomplicated clinical courses [35].

#### 2.2.2. Kidney Injury Molecule-1 (KIM-1)

After ischemic or toxic damage, renal proximal tubules release kidney injury molecule-1 (KIM-1), a type I transmembrane protein [42,43]. The role of KIM-1, associated with tubulointerstitial inflammation and fibrosis, has not been fully elucidated; however, its role is related to apoptosis and the regeneration of injured tubules, participating in the restoration of tubular structure and function. Moreover, macrophages other than proximal tubular epithelial cells are implicated in tubular repair [44].

This biomarker has been evaluated in the same pathological fields of NGAL, from cardiac surgery to AKI related to nephrotoxic medications. However, contrasting data are available in the literature, without a clear indication to use this biomarker alone in the pediatric setting, but it is hypothesized that a panel of biomarkers with KIM-1 can reveal damage in proximal renal tubules.

#### 2.2.3. Cystatin C

Serum cystatin C (CC) is entirely removed from the blood by glomerular filtration because of its low molecular weight and positive charge, reflecting a better GFR than those of other low-molecular-weight proteins, including creatinine. It is then reabsorbed and catabolized in the proximal renal tubule [45]. Infections, inflammatory diseases and liver diseases do not alter filtration, suggesting that CC represents a marker of GFR more accurately than serum creatinine [46]. It has been demonstrated that sCr and CC levels are significantly higher in AKI patients, correlating with each other [47]. There is a strong correlation between serum CC concentrations and sCr levels in pediatric AKI patients, revealing a better diagnostic profile of serum CC than that of sCr [48]. Similarly, in a prospective, observational study, serum CC and βeta-2 microglobulin (β2MG) had more sensitive and specific properties than those of sCr in detecting AKI in critically ill children [49,50]. Moreover, serum CC levels detected AKI early in preterm neonates with respiratory distress syndrome (RDS) [51].

Reference values for CC in preterm infants were established and compared with sCR, and they ranged from 1.25 to 2.84 mg/L at the first day of life, significantly decreasing after 3 days [50].

CC concentrations in children reach adult values by the age of 1 year (range, 0.50–1.27 mg/L; adult range, 0.51–0.98), whereas higher CC levels are assessed during the first year of life, reflecting the immaturity of the kidneys (<1 year: 0.59–1.97 mg/L) [52].

CC levels in healthy Turkish neonates were determined to be 1.41 mg/L, 1.22 mg/L and 1.21 mg/L for neonates born in gestational weeks 28–32 and 33–36 and in neonates born after gestational week 37, respectively [53].

Serum CC level was found to have a statistically significant association with AKI development in preterm neonates with respiratory distress syndrome (RDS) [51].

In more than fifty thousand Chinese neonates, cystatin C-related criteria, independent from gestational age and birth weight, defined AKI by a serum Cys-C of ≥2.2 mg/L or when an increase in Cys-C of ≥25% occurred, with a positive correlation with an increased risk of in-hospital mortality [54].

However, contrasting data refer also to CC, as assessed in neonates after cardiac surgery in whom, despite a significant difference in CC levels from baseline and after 13 h, only urine output was independently associated with AKI [55].

Further studies enrolling more homogenous pediatric and neonatal populations are needed to understand the role of CC as a precocious renal biomarker of AKI.

#### 2.2.4. Interleukin-18

Interleukin (IL)-18, a pro-inflammatory cytokine that stimulates interferon-gamma production in T cells and natural killer cells, is upregulated in patients with AKI. In particular, during ischemic acute renal damage, a precursor of IL-18 is activated by caspase-1-mediated conversion and released by tubular cells, representing a marker of proximal tubular injury in ischemic acute tubular necrosis [56]. High urinary IL-18 levels are characterized by ischemia, acute tubular damage and delayed graft function compared with other renal diseases, allowing for the early diagnosis of AKI [57]. Other data suggest a prognostic role of urine IL-18 in patients with RDS in intensive care, reflecting the mortality risk [58]. Similarly, urinary IL-18 predicted the severity of AKI and was an independent predictor of mortality in non-septic critically ill children, revealing a better diagnostic profile than that of sCr [59,60].

#### 2.2.5. TIMP2 × IGFBP7

Tissue inhibitor of metalloproteinase-2 (TIMP-2) and insulin-like growth factor-binding protein 7 (IGFBP7) are two urinary G1 cell cycle arrest biomarkers for the early diagnosis of kidney damage. Following injury, renal tubular cells enter a period of G1 cell cycle arrest, leading to cellular repair, cell death or cellular senescence [61,62].

The product of these urinary markers [TIMP-2] × [IGFBP7] performed better than any other biomarker in detecting AKI according to the KDIGO criteria, including neonatal and pediatric patients [63]. Pediatric studies using these biomarkers to predict AKI are limited.

In post-cardiac surgery, the diagnostic properties of these markers were comparable to NGAL, with better profiles assessed at 4 h and 12 h post-cardio-pulmonary bypass for predicting AKI [64,65].

Moreover, severe AKI was assessed in 230 patients undergoing complex cardiac surgery using [TIMP-2] × [IGFBP7], predicting late severe AKI but not precocious AKI, as early as 2 h after the start of cardiopulmonary bypass [66]. [TIMP-2] × [IGFBP7] was tested in critically ill neonates to predict AKI and severe AKI, revealing its independent predictive role, independent of sex, illness severity and gestational age, in the heterogeneous critical neonatal population [67].

Further prospective studies are required in larger pediatric populations to evaluate the role of these biomarkers in different subtypes of AKI.

## 3. Risk Factors for AKI in Neonatal Intensive Care

### 3.1. Prematurity and Low Birth Weight

Preterm neonates represent a population at a high risk for AKI due to a potential incomplete and abnormal process of nephrogenesis, resulting in decreased nephron mass [68]. The reduction in renal mass in the preterm became the subject of follow-up studies on cohorts of preterm infants. These studies identifyied subjects at risk, and implemented preventive interventions and revealed the connection between low birth weight, decreased nephron mass and blood hypertension [69,70].

According to Barker’s hypothesis, low-birth-weight (LBW) babies could have a reduced nephron-related mass, altering the sodium transport and metabolism in the glomeruli, with a high risk of developing blood hypertension [71]. However, no guidelines suggest the prescription of antihypertensive drugs for LBW patients with a risk of hypertension. In addition, neonates could have reduced renal blood flow associated with low GFR, increasing from 10 to 20 mL/min/1.73 m^2^ during the first week of life to 120 mL/min/1.73 m^2^ in the first year, reaching adult levels by 2 years of age [72,73,74].

The proximal tubule reabsorbs various substances filtered at the glomerular level (amino acids, bicarbonates and glucose) through active and passive transport mechanisms. These transport systems are immature in preterm infants, causing metabolic acidosis and glycosuria; the tubular phosphorus reabsorption system is more active in newborns than that in adults and can therefore lead to hyperphosphatemia [75]. Hyperkalemia is frequently observed in preterm newborns due to the immaturity of sodium and potassium transport systems and the regulation of levels in the collecting duct. Moreover, intrauterine growth retardation can alter kidney maturation, interfering with nephrogenesis and resulting in a reduction in kidney size compared to reference values [76]. Chen revealed that oliguric AKI had a higher mortality risk than that of non-oliguric AKI, independent of sCr levels and the severity of AKI. In particular, prenatal small-for-gestational-age and perinatal and postnatal adverse events were often associated with oliguric AKI, and nephrotoxin exposures caused non-oliguric AKI [77].

Mazhaeri confirmed this link between AKI, prematurity and LWB in association with low Apgar scores, mechanical ventilation and sepsis [78].

### 3.2. Sepsis

Sepsis-related AKI (sAKI) represents one of the main mortality risks in the NICU, of around 70% [79]. Neonatal sepsis is frequently assessed in preterm newborns, secondary to pathogens acquired after birth, and is defined as early (ES) or late (LS) sepsis if diagnosis occurs within or after 72 h of birth, respectively [80]. Neonatal sepsis is inversely related to birth weight and gestational age, with a high prevalence in very-low-birth-weight neonates [81] and with gestational age (GA) < 28 weeks [82].

Sepsis therapy is based on the administration of drugs that can have nephrotoxic action and worsen the renal damage caused by hypotension and hypoperfusion [83]. However, contrasting data are reported about the subtypes of sepsis, underlining that Munyendo revealed that AKI complicates the clinical course of neonates with LS, whereas Holda demonstrated a high incidence of AKI in ES patients [84,85].

Moreover, primiparity and the presence of maternal fever within a week of delivery are maternal factors associated with AKI, as well as peripartum sepsis and exposure to antibiotics in the immediate peripartum period [86].

### 3.3. Post-Neonatal Asphyxia, Respiratory Distress and Mechanical Ventilation

Neonatal asphyxia represents a risk factor for AKI, closely related to its severity, and it induces multiorgan dysfunction, including renal failure, due to ischemic processes.

A wide range of AKI prevalence, between 30 and 56% [87,88] due to different definitions, involves neonates with asphyxia, representing, however, a non-negligible cause of AKI, with incidence also higher than that related to sepsis, according to some epidemiological reports [89,90]. El-Kalioby reported other epidemiological data, including perinatal asphyxia (59.5%) as a risk factor of AKI, independent from RDS, shock, prematurity, sepsis and nephrotoxic drugs [91]. Similarly, mechanical ventilation (MV) represents another risk of AKI due to hemodynamic factors or ventilator-induced lung injury, inducing pulmonary and systemic inflammation [92,93]. Moreover, these data were confirmed in a retrospective study in which invasive MV, associated with a lower GA and birth weight, were independent risk factors for AKI in newborns [94]. Underlining the potential role of systemic inflammation and several inflammatory pathways involved in this acute lung–kidney crosstalk, the relation between MV and AKI remains unclear.

### 3.4. Nephrotoxic Drugs

Nephrotoxic drugs represent a frequent cause of AKI in critically ill neonates with long-term complications, leading to potential chronic kidney disease (CKD).

Of neonates, 75% are treated with at least one nephrotoxic medication, i.e., aminoglycosides, during the first postnatal week in the NICU, with a close relationship with AKI [95]. Behind the high rate of treated patients, this cause of AKI is potentially preventable. Nephrotoxic drugs can be avoided, their prescription can be modulated according to kidney function, or concomitant comorbidities that expose neonates in the NICU at a high risk of AKI can be evaluated. The link between drugs and acute renal dysfunction remains understudied, having only been researched by few studies, revealing, especially in premature neonates, that the cumulative exposition of drugs is closely related to AKI risk [96,97,98,99,100].

Nephrotoxicity depends on several factors, including age, comorbidities, the dosage of the drug and concomitant medications, considering that more than 80% of neonates often receive one or more nephrotoxic drugs, such as vancomycin or gentamicin [101,102]. Infants with very low birth weights (VLBWs) are the most studied population in the NICU, revealing acute interstitial nephritis and acute tubular necrosis as the main physiopathological mechanisms of renal injury [103]. The effects of these processes often lead to hypokalemia due to a reversible renal tubular waste of potassium, also including phosphate and calcium, with full recovery within 1–2 weeks after the discontinuation of the drug [96].

Antibiotics can induce nephrotoxicity with a decreased risk: carbapenems > cephalosporins > penicillins > monobactams. Conversely, third-generation cephalosporins do not induce renal damage [104].

Behind bacterial infections, VLBW infants are characterized by a non-negligible risk of systemic fungal infection. When *Candida* sp. represents the major pathogen, amphotericin B is the treatment of choice, highlighting that the encapsulated liposome (AmBisome) forms do not induce nephrotoxic effects [105,106].

A preventive strategy to reduce the risk of nephrotoxicity related to drug administration was developed by Goldstein, who created the Nephrotoxic Injury Negated by Just-in-time Action (NINJA) program, identifying pediatric patients at a high risk for AKI according to sCr monitoring [107]. Similarly, Stoops applied the NINJA program to the NICU, creating the ‘Baby NINJA’ and reducing nephrotoxic medication exposure and AKI rates in the NICU [108]. These programs, associated with electronic alerts, could help clinicians reduce nephrotoxic medication exposures and AKI events.

### 3.5. Patent Ductus Arteriosus and Cardiac Surgery

Persistently patent ductus arteriosus (PDA) in preterm infants induces hemodynamic instability due to a left-to-right shunt, leading to chronic lung disease and AKI, necrotizing enterocolitis with a high risk of perforation [109].

PDA can cause a condition of relative hypovolemia and hypoperfusion with an increase in the synthesis of vasodilatory prostaglandins in an attempt to compensate for the hemodynamic effects of the duct. In this condition, non-steroidal anti-inflammatory drug (NSAD) administration reduces this vasodilation effect, but it may worsen renal perfusion, with consequent renal injury and damage [110]. Although it is traditional to manage PDA with NSAD, guidelines for therapeutical management are lacking, with a high risk of adverse events related to these drugs [111,112,113]. In particular, indomethacin can cause transient renal failure due to a reduction in prostaglandins, which act at the renal level, causing vasodilation mediated by the inhibition of vasopressin [74]. Ibuprofen inhibits the vasodilator effects of prostaglandins on the afferent glomerular arterioles, reducing the GFR and diuresis [114]. However, it induces fewer adverse effects on renal hemodynamics, if compared to indomethacin, representing the first choice for PDA treatment in preterm infants [115,116].

Cardiac surgery represents one of the most frequent causes of AKI in neonates, with a high risk of a longer stay and mortality [117]. Different mechanisms are related to this type of surgery for AKI, such as a prolonged cross-clamp time, a prolonged MV, a longer time to reach a negative fluid balance and higher inotropic support [118].

## 4. Recurrent AKI and Transition to CKD

CKD represents a potential long-term complication in neonates who suffer from AKI during NICU stays [119,120]. Moreover, AKI events can also relapse after an initial resolution before discharge from the NICU, defined as recurrent AKI (rAKI) according to the KDIGO definition.

rAKI incidence remains largely unknown, ranging between 7% and 24% [2,121,122,123] due to few single-center studies that have assessed it in neonates.

GA and LBW are risk factors for rAKI, which is related to a longer stay in the NICU and to increased mortality if compared to patients who suffer from only one AKI event [121]. rAKI is also related to a prolonged time of mechanical ventilation [123]. These data, obtained from these single-center studies, were confirmed by the multicentric trial AWAKEN, which analyzed more than two thousand critically ill neonates, revealing a higher incidence of rAKI among neonates with the youngest GAs and lowest birth weights; rAKI also occurred in patients with severe first AKI episodes with low Apgar scores and if respiratory failure, sepsis or congenital heart disease occurred [124].

rAKI represents a prognostic factor that can be used to predict potential evolution to chronic kidney disease (CKD), as assessed both in adults and in childhood [5,125,126,127,128,129]. The progressive loss of nephrons, leading to initial hyperfiltration, associated with endothelial dysfunction, represents the final effect of maladaptive repair involving the kidneys, with pro-inflammatory processes, tubulo-interstitial fibrosis and glomerulosclerosis [119].

A literature review showed a wide range (10–70%) of CKD rates after AKI in children [130,131] due to the lack of trials analyzing outcomes with an insufficient follow-up time, requiring an analysis of CKD prevalence during adulthood. In particular, birth weight is negatively related to sCr levels in adults born very preterm. Similarly, intrauterine growth retardation (IUGR) is associated with CKD in subjects born very prematurely, suggesting that IUGR represents a non-negligible risk factor for renal failure, increasing systemic arterial stiffness and mean blood pressure [132,133].

These hypotheses were confirmed by Franke, who highlighted that, in more than four hundred children, SGA and prematurity represent risk factors for CKD [134]. Other studies have revealed that proteinuria, albuminuria, a loss of renal mass and nephrocalcinosis characterize preterm infants with extreme LBW and AKI over an 18-year period [76,135].

## 5. Conclusions

AKI complicates the clinical course in critically ill neonates in the NICU, inducing prolonged hospitalization and a high mortality risk. Accurate and precocious diagnosis of AKI is required to evaluate the prevalence of AKI, the risk factors and a personalized therapeutical strategy and to create follow-up plans for infants at risk of rAKI and transitioning to CKD. Observational studies based on prospective physiological findings, including a large number of premature and term babies stratified according to gestational weeks and risk factors, are required.

To achieve these goals, new biomarkers should be tested and validated in neonatal and pediatric populations. However, to date, only cystatin C is suitable for clinical use, whereas all reported renal biomarkers are not commonly used in clinical practice.

Large multicenter studies with long-term follow-ups are needed to understand the physiopathological mechanisms and the risk of CKD in patients with AKI events that occur during childhood.

## References

[1] Blinder J.J., Asaro L.A., Wypij D., Selewski D.T., Agus M.S.D., Gaies M., Ferguson M.A. (2017). Acute kidney injury after pediatric cardiac surgery: A secondary analysis of the safe pediatric euglycemia after cardiac surgery trial. Pediatr. Crit. Care Med..

[2] Carmody J.B., Swanson J.R., Rhone E.T., Charlton J.R. (2014). Recognition and reporting of AKI in very low birth weight infants. Clin. J. Am. Soc. Nephrol..

[3] Walker M.W., Clark R.H., Spitzer A.R. (2011). Elevation in plasma creatinine and renal failure in premature neonates without major anomalies: Terminology, occurrence and factors associated with increased risk. J. Perinatol..

[4] Jetton J.G., Boohaker L.J., Sethi S.K., Wazir S., Rohatgi S., Soranno D.E., Chishti A.S., Woroniecki R., Mammen C., Swanson J.R. (2017). Incidence and outcomes of neonatal acute kidney injury (AWAKEN): A multicentre, multinational, observational cohort study. Lancet Child. Adolesc. Health.

[5] Chaturvedi S., Ng K.H., Mammen C. (2017). The path to chronic kidney disease following acute kidney injury: A neonatal perspective. Pediatr. Nephrol..

[6] Acute Kidney Injury Work Group (2012). KDIGO clinical practice guideline for acute kidney injury. Kidney Int. Suppl..

[7] Sutherland S.M., Byrnes J.J., Kothari M., Longhurst C.A., Dutta S., Garcia P., Goldstein S.L. (2015). AKI in hospitalized children: Comparing the pRIFLE, AKIN, and KDIGO defnitions. Clin. J. Am. Soc. Nephrol..

[8] Kaur S., Jain S., Saha A., Chawla D., Parmar V.R., Basu S., Kaur J. (2011). Evaluation of glomerular and tubular renal function in neonates with birth asphyxia. Ann. Trop. Paediatr..

[9] Levey A.S., Bosch J.P., Lewis J.B., Greene T., Rogers N., Roth D. (1999). A more accurate method to estimate glomerular filtration rate from serum creatinine: A new prediction equation. Modification of Diet in Renal Disease Study Group. Ann. Intern. Med..

[10] Makris K., Spanou L. (2016). Acute Kidney Injury: Diagnostic approaches and controversies. Clin. Biochem. Rev..

[11] Waikar S.S., Betensky R.A., Emerson S.C., Bonventre J.V. (2012). Imperfect gold standards for kidney injury biomarker evaluation. J. Am. Soc. Nephrol..

[12] Colantonio D.A., Kyriakopoulou L., Chan M.K., Daly C.H., Brinc D., Venner A.A., Pasic M.D., Armbruster D., Adeli K. (2012). Closing the gaps in pediatric laboratory reference intervals: A CALIPER database of 40 biochemical markers in a healthy and multiethnic population of children. Clin. Chem..

[13] Xu X., Nie S., Zhang A., Jianhua M., Liu H.P., Xia H., Xu H., Liu Z., Feng S., Zhou W. (2018). A New Criterion for Pediatric AKI Based on the Reference Change Value of Serum Creatinine. JASN.

[14] Zeng J., Miao H., Jiang Z., Zhang Y., Guo X., Chen Q., Wan Y., Ji P., Xie G., Li H. (2022). Pediatric Reference Change Value Op-timized for Acute Kidney Injury: Multicenter Retrospective Study in China. Pediatr. Crit. Care Med..

[15] Goldstein S.L. (2018). A new pediatric AKI definition: Implications of trying to build the perfect mousetrap. J. Am. Soc. Nephrol..

[16] Kuai Y., Li M., Chen J., Jiang Z., Bai Z., Huang H., Wei L., Liu N., Li X., Lu G. (2022). Comparison of diagnostic criteria for acute kidney injury in critically ill children: A multicenter cohort study. Crit Care.

[17] Thomas M.E., Blaine C., Dawnay A., Devonald M.A., Ftouh S., Laing C., Latchem S., Lewington A., Milford D.V., Ostermann M. (2015). The definition of acute kidney injury and its use in practice. Kidney Int..

[18] Miall L.S., Henderson M.J., Turner A.J., Brownlee K.G., Brocklebank J.T., Newell S.J., Allgar V.L. (1999). Plasma creatinine rises dramatically in the first 48 hours of life in preterm infants. Pediatrics.

[19] Coulthard M.G., Hey E.N., Ruddock V. (1985). Creatinine and urea clearances compared to inulin clearance in preterm and mature babies. Early Hum. Dev..

[20] Goldstein S.L., Currier H., Graf C.D., Cosio C.C., Brewer E.D., Sachdeva R. (2001). Outcome in children receiving continuous venovenous hemofiltration. Pediatrics.

[21] Macedo E., Bouchard J., Soroko S.H., Chertow G.M., Himmelfarb J., Ikizler T.A., Paganini E.P., Mehta R.L., Program to Improve Care in Acute Renal Disease Study (2010). Fluid accumulation, recognition and staging of acute kidney injury in critically-ill patients. Crit Care.

[22] Askenazi D. (2011). Evaluation and management of critically ill children with acute kidney injury. Curr. Opin. Pediatr..

[23] Branagan A., Costigan C.S., Stack M., Slagle C., Molloy E.J. (2022). Management of Acute Kidney Injury in Extremely Low Birth Weight Infants. Front. Pediatr..

[24] Devarajan P. (2008). Emerging urinary biomarkers in the diagnosis of acute kidney injury. Expert Opin. Med. Diagn..

[25] Mishra J., Ma Q., Prada A., Mitsnefes M., Zahedi K., Yang J., Barasch J., Devarajan P. (2003). Identification of neutrophil gelatinase-associated lipocalin as a novel early urinary biomarker for ischemic renal injury. J. Am. Soc. Nephrol..

[26] Parikh C.R., Mishra J., Thiessen-Philbrook H., Dursun B., Ma Q., Kelly C., Dent C., Devarajan P., Edelstein C.L. (2006). Urinary IL-18 is an early predictive biomarker of acute kidney injury after cardiac surgery. Kidney Int..

[27] Han W.K., Bailly V., Abichandani R., Thadhani R., Bonventre J.V. (2002). Kidney Injury Molecule-1 (KIM-1): A novel biomarker for human renal proximal tubule injury. Kidney Int..

[28] Liangos O., Perianayagam M.C., Vaidya V.S., Han W.K., Wald R., Tighiouart H., MacKinnon R.W., Li L., Balakrishnan V.S., Pereira B.J. (2007). Urinary N-acetyl-beta-(D)-glucosaminidase activity and kidney injury molecule-1 level are associated with adverse outcomes in acute renal failure. J. Am. Soc. Nephrol..

[29] Trof R.J., Di Maggio F., Leemreis J., Groeneveld A.B. (2006). Biomarkers of acute renal injury and renal failure. Shock.

[30] Mishra J., Dent C., Tarabishi R., Mitsnefes M.M., Ma Q., Kelly C., Ruff S.M., Zahedi K., Shao M., Bean J. (2005). Neutrophil gelatinaseassociated lipocalin (NGAL) as a biomarker for acute renal injury after cardiac surgery. Lancet.

[31] Suchojad A., Tarko A., Smertka M., Majcherczyk M., Brzozowska A., Wroblewska J., Maruniak-Chudek I. (2015). Factors limiting usefulness of serum and urinary NGAL as a marker of acute kidney injury in preterm newborns. Ren. Fail..

[32] Bolignano D., Coppolino G., Romeo A., Lacquaniti A., Buemi M. (2010). Neutrophil gelatinase-associated lipocalin levels in chronic haemodialysis patients. Nephrology.

[33] Chen W.C., Lin H.S., Tsai F.J., Li C.W. (2001). Effects of Tamm-Horsfall protein and albumin on the inhibition of free radicals. Urol. Int..

[34] Zappitelli M., Washburn K.K., Arikan A.A., Loftis L., Ma Q., Devarajan P., Parikh C.R., Goldstein S.L. (2007). Urine neutrophil gelatinase-associated lipocalin is an early marker of acute kidney injury in critically ill children: A prospective cohort study. Crit. Care.

[35] Huynh T.K., Bateman D.A., Parravicini E., Lorenz J.M., Nemerofsky S.L., Sise M.E., Bowman T.M., Polesana E., Barasch J.M. (2009). Reference Values of Urinary Neutrophil Gelatinase-Associated Lipocalin in Very Low Birth Weight Infants. Pediatr. Res..

[36] Lavery A.P., Meinzen-Derr J.K., Anderson E., Ma Q., Bennett M.R., Devarajan P., Schibler K.R. (2008). Urinary NGAL in premature infants. Pediatr. Res..

[37] El-Achkar T.M., Wu X.R., Rauchman M., McCracken R., Kiefer S., Dagher P.C. (2008). Tamm-Horsfall protein protects the kidney from ischemic injury by decreasing inflammation and altering TLR4 expression. Am. J. Physiol. Renal. Physiol..

[38] Kayaaltı S., Kayaaltı O., Aksebzeci B.H. (2018). Relationship between Neutrophil Gelatinase-associated Lipocalin and Mortality in Acute Kidney Injury. Turk. J. Intensiv. Care.

[39] Merve I., Mahmut O.K., Amir N., Alper E., Tugba M.P., Ramazan Y., Murat K.D. (2023). Acute Kidney Injury Is Associated with Higher Serum Cys-C and NGAL Concentrations, and Risk of Mortality in Premature Calves with Respiratory Distress Syndrome. Animals.

[40] Hirsch R., Dent C., Pfriem H., Allen J., Beekman R.H., Ma Q., Dastrala S., Bennett M., Mitsnefes M., Devarajan P. (2007). NGAL is an early predictive biomarker of contrast-induced nephropathy in children. Pediatr. Nephrol..

[41] Stoops C., Gavigan H., Krallman K., Anderson N., Griffin R., Slagle C., House S., Goldstein S.L., Askenazi D.J. (2024). The Utility of Urinary NGAL as an Alternative for Serum Creatinine to Detect Acute Kidney Injury in Infants Exposed to Nephrotoxic Medications in the Neonatal Intensive Care Unit. Neonatology.

[42] van Timmeren M.M., van den Heuvel M.C., Bailly V., Bakker S.J.L., van Goor H., Stegeman C.A. (2007). Tubular kidney injury molecule-1 (KIM-1) in human renal disease. J. Pathol..

[43] Cherian S., Crompton C.H. (2005). Partial hypoxanthine-guanine phosphoribosyltransferase deficiency presenting as acute renal failure. Pediatr. Nephrol..

[44] Ai Ing L., Sydney C.W., Tang K.N.L., Joseph C.K. (2013). Leung Kidney injury molecule-1: More than just an injury marker of tubular epithelial cells?. J. Cell Physiol..

[45] Treiber M., Pecovnik-Balon B., Gorenjak M. (2006). Cystatin C versus creatinine as a marker of glomerular filtration rate in the newborn. Wien. Klin. Wochenschr..

[46] Finney H., Newman D.J., Price C.P. (2000). Adult reference for serum cystatin C, creatinine and predicted creatinine clearance. Ann. Clin. Biochem..

[47] Tarif N., Alwakeel J.S., Mitwalli A.H., Durdana H., Memon N.A., Askar A., Chaudhary A.R., Isnani A.C. (2008). Serum cystatin C as a marker of renal function in patients with acute renal failure. Saudi J. Kidney Dis. Transplant..

[48] Villa P., Jiménez M., Soriano M.C., Manzanares J., Casasnovas P. (2005). Serum cystatin C concentration as a marker of acute renal dysfunction in critically ill patients. Crit. Care.

[49] Herrero-Morín J.D., Málaga S., Fernández N., Rey C., Diéguez M.A., Solís G., Concha A., Medina A. (2007). Cystatin C and beta2-microglobulin: Markers of glomerular filtration in critically ill children. Crit. Care.

[50] Armanji D., Yurdakök M., Canpolat F.E., Korkmaz A., Yiğit S., Tekinalp G. (2008). Determination of reference values for plasma cystatin C and comparison with creatinine in premature infants. Pediatr. Nephrol..

[51] Elmas A.T., Tabel Y., Elmas O.N. (2013). Serum cystatin C predicts acute kidney injury in preterm neonates with respiratory distress syndrome. Pediatr. Nephrol..

[52] Finney H., Newman D.J., Thakkar H., Fell J.M.E., Price C.P. (2000). Reference ranges for plasma cystatin C and creatinine measurements in premature infants, neonates, and older children. Arch. Dis. Child.

[53] Dorum S., Silfeler I., Dorum B.A., Silfeler D.B., Canbak Y., Say A. (2012). Reference values of serum cystatin-C for full-term and preterm neonates in Istanbul. Indian J. Pediatr..

[54] Xu X., Nie S., Xu H., Liu B., Weng J., Chen C., Liu H., Yang Q., Li H., Kong Y. (2023). Detecting Neonatal AKI by Serum Cystatin C. J. Am. Soc. Nephrol..

[55] Abadeer M., Swartz M.F., Martin S.D., Groves A.M., Kent A.L., Schwartz G.J., Brophy P., Alfieris G.M., Cholette J.M. (2023). Using Serum Cystatin C to Predict Acute Kidney Injury Following Infant Cardiac Surgery. Pediatr. Cardiol..

[56] Melnikov V.Y., Ecder T., Fantuzzi G., Siegmund B., Lucia M.S., Dinarello C.A., Schrier R.W., Edelstein C.L. (2001). Impaired IL-18 processing protects caspase-1-deficient mice from ischemic acute renal failure. J. Clin. Investig..

[57] Parikh C.R., Jani A., Vyacheslav Y., Melnikov S.F., Edelstein C.L. (2004). Urinary interleukin-18 is a marker of human acute tubular necrosis. Am. J. Kidney Dis..

[58] Parikh C.R., Abraham E., Ancukiewicz M., Edelstein C.L. (2005). Urine IL-18 is an early diagnostic marker for acute kidney injury and predicts mortality in the intensive care unit. J. Am. Soc. Nephrol..

[59] Washburn K.K., Zappitelli M., Arikan A.A., Loftis L., Yalavarthy R., Parikh C.R., Edelstein C.L., Goldstein S.L. (2008). Urinary interleukin-18 is an acute kidney injury biomarker in critically ill children. Nephrol. Dial. Transplant..

[60] Leslie J.A., Meldrum K.K. (2008). The role of interleukin-18 in renal injury. J. Surg. Res..

[61] Kashani K., Al-Khafaji A., Ardiles T., Artigas A., Bagshaw S.M., Bell M., Bihorac A., Birkhahn R., Cely C.M., Chawla L.S. (2013). Discovery and validation of cell cycle arrest biomarkers in human acute kidney injury. Crit. Care.

[62] Lacquaniti A., Ceresa F., Campo S., Barbera G., Caruso D., Palazzo E., Patanè F., Monardo P. (2023). Acute Kidney Injury and Sepsis after Cardiac Surgery: The Roles of Tissue Inhibitor Metalloproteinase-2, Insulin-like Growth Factor Binding Protein-7, and Mid-Regional Pro-Adrenomedullin. J. Clin. Med..

[63] Westhoff J.H., Tönshoff B., Waldherr S., Pöschl J., Teufel U., Westhoff T.H., Fichtner A. (2015). Urinary Tissue Inhibitor of Metalloproteinase-2 (TIMP-2) • Insulin-Like Growth Factor-Binding Protein 7 (IGFBP7) Predicts Adverse Outcome in Pediatric Acute Kidney Injury. PLoS ONE.

[64] Meersch M., Schmidt C., Van Aken H., Rossaint J., Görlich D., Stege D., Malec E., Januszewska K., Zarbock A. (2014). Validation of cell-cycle arrest biomarkers for acute kidney injury after pediatric cardiac surgery. PLoS ONE.

[65] Gist K.M., Goldstein S.L., Wrona J., Alten J.A., Basu R.K., Cooper D.S., Soranno D.E., Duplantis J., Altmann C., Gao Z. (2017). Kinetics of the cell cycle arrest biomarkers (TIMP-2*IGFBP-7) for prediction of acute kidney injury in infants after cardiac surgery. Pediatr. Nephrol..

[66] Tao Y., Heskia F., Zhang M., Qin R., Kang B., Chen L., Wu F., Huang J., Brengel-Pesce K., Chen H. (2022). Evaluation of acute kidney injury by urinary tissue inhibitor metalloproteinases-2 and insulin-like growth factor-binding protein 7 after pediatric cardiac surgery. Pediatr. Nephrol..

[67] Chen J., Sun Y., Wang S., Dai X., Huang H., Bai Z., Li X., Wang J., Li Y. (2020). The effectiveness of urinary TIMP-2 and IGFBP-7 in predicting acute kidney injury in critically ill neonates. Pediatr. Res..

[68] Chambers J.M., Wingert R.A. (2020). Advances in understanding vertebrate nephrogenesis. Tissue Barriers.

[69] Kandasamy Y., Smith R., Wright I.M., Lumbers E.R. (2012). Relationships between glomerular filtration rate and kidney volume in low-birth-weight neonates. J. Nephrol..

[70] Bagby S.P. (2009). Developmental origins of renal disease: Should nephron protection begin at birth?. Clin. J. Am. Soc. Nephrol..

[71] Felix J., Nihal T. (2021). Barker Hypothesis and Hypertension. Front. Public Health.

[72] Askenazi D.J., Ambalavanan N., Goldstein S.L. (2009). Acute kidney injury in critically ill newborns: What do we know? What do we need to learn?. Pediatr. Nephrol..

[73] Drukker A., Guignard J.P. (2002). Renal aspects of the term and preterm infant: A selective update. Curr. Opin. Pediatr..

[74] Pai V.B., Sakadjian A., Puthoff T.D. (2008). Ibuprofen lysine for the prevention and treatment of patent ductus arteriosus. Pharmacotherapy.

[75] Pamela I., Li G.L., Hurst H.A., Herrera I.S., Xu K., Rao M., Bateman D.A., Al-Awqati D., D’Agati V.D., Costantini F. (2023). Low nephron endowment increases susceptibility to renal stress and chronic kidney disease. JCI Insight.

[76] Abitbol C.L., Bauer C.R., Montané B., Chandar J., Duara S., Zilleruelo G. (2003). Long-term follow-up of extremely low birth weight infants with neonatal renal failure. Pediatr. Nephrol..

[77] Chen C.C., Chu C.H., Lin Y.C., Wang S.T., Huang C.C. (2023). Preceding risks and mortality outcomes of different neonatal acute kidney injury in preterm infants. Pediatr. Res..

[78] Mazaheri M., Rambod M. (2019). Risk factors analysis for acute kidney injury in the newborn infants: Predictive strategies. Iran. J. Kidney Dis..

[79] Cai C., Qiu G., Hong W., Shen Y., Gong X. (2020). Clinical effect and safety of continuous renal replacement therapy in the treatment of neonatal sepsis-related acute kidney injury. BMC Nephrol..

[80] Celik I.H., Hanna M., Canpolat F.E., Pammi M. (2022). Diagnosis of Neonatal Sepsis: The Past, Present and Future. Pediatr. Res..

[81] Barbosa J.D.S., Silva Júnior G.B.D., Meneses G.C., Martins A.M.C., Daher E.F., Machado R.P.G., Lemes R.P.G. (2022). Use of non-conventional biomarkers in the early diagnosis of acute kidney injury in preterm newborns with sepsis. J. Bras. Nefrol..

[82] Tsai M.H., Hsu J.F., Chu S.M., Lien R., Huang H.R., Chiang M.C., Fu R.H., Lee C.W., Huang Y.C. (2014). Incidence, Clinical Characteristics and Risk Factors for Adverse Outcome in Neonates with Late-onset Sepsis. Pediatr. Infect. Dis. J..

[83] Little D.C., Pratt T.C., Blalock S.E., Krauss D.R., Cooney D.R., Custer M.D. (2003). Patent ductus arteriosus in micropreemies and full-term infants: The relative merits of surgical ligation versus indomethacin treatment. J. Pediatr. Surg..

[84] Munyendo C., Admani B., Mburugu P., Simba J., Lusweti B., Gachara N., Laving A. (2023). Prevalence of acute kidney injury and its characteristics among neonates with suspected sepsis in a tertiary hospital in Kenya. Afr. Health Sci..

[85] Holda A.M., Mehariya K.M., Patel P., Patel P. Study of Effect of Neonatal Septicemia on Renal Function. http://www.ijsr.net/archive/v3i12/U1VCMTQxMDg2.pdf.

[86] Cataldi L., Leone R., Moretti U., De Mitri B., Fanos V., Ruggeri L., Sabatino G., Torcasio F., Zanardo V., Attardo G. (2005). Potential risk factors for the development of acute renal failure in preterm newborn infants: A case-control study. Arch. Dis. Child. Fetal Neonatal.

[87] Aggarwal A., Kumar P., Chowdhary G., Majumdar S., Narang A. (2005). Evaluation of renal functions in asphyxiated newborns. J. Trop. Pediatr..

[88] Durkan A.M., Alexander R.T. (2011). Acute kidney injury post neonatal asphyxia. J. Pediatr..

[89] Gharehbaghi M.M., Peirovifor A. (2007). Evaluating causes of acute renal failure in newborn intants. Pak. J. Med. Sci..

[90] Mortazavi F., Hosseinpour Sakha S., Nejati N. (2009). Acute kidney failure in neonatal period. Iran. J. Kidney Dis..

[91] El-Kalioby M., Khashana A., Kamel N., Hennawi S. (2022). Causes of Neonatal Acute Renal Injury during Critical Illnesses. Saudi J. Kidney Dis. Transplant..

[92] Viswanathan S., Manyam B., Azhibekov T., Mhanna M.J. (2012). Risk factors associated with acute kidney injury in extremely low birth weight (ELBW) infants. Pediatr. Nephrol..

[93] Kuiper J.W., Groeneveld A.B., Slutsky A.S., Plötz F.B. (2005). Mechanical ventilation and acute renal failure. Crit. Care Med..

[94] Fan Y., Ye J., Qian L., Zhao R., Zhang N., Xue L., Qiao L., Jiang L. (2019). Risk factors and outcomes of acute kidney injury in ventilated newborns. Ren. Fail..

[95] Steflik H.J., Charlton J.R., Briley M., Selewski D.T., Gist K.M., Hanna M.H., Askenazi D., Griffin R. (2023). Neonatal Kidney Collaborative*. Neonatal Nephrotoxic Medication Exposure and Early Acute Kidney Injury: Results from the AWAKEN Study. J. Perinatol..

[96] Barhight M., Altaye M., Gist K.M., Isemann B., Goldstein S.L., Akinbi H. (2017). Nephrotoxic Medications and Associated Acute Kidney Injury in Very Low Birth Weight Infants. J. Clin. Nephrol. Res..

[97] Murphy H.J., Thomas B., Van Wyk B., Tierney S.B., Selewski D.T., Jetton J.G. (2020). Nephrotoxic medications and acute kidney injury risk factors in the neonatal intensive care unit: Clinical challenges for neonatologists and nephrologists. Pediatr. Nephrol..

[98] Mohamed T.H., Abdi H.H., Magers J., Prusakov P., Slaughter J.L. (2022). Nephrotoxic medications and associated acute kidney injury in hospitalized neonates. J. Nephrol..

[99] Hsieh E.M., Hornik C.P., Clark R.H., Laughon M.M., Benjamin D.K., Smith P.B. (2014). Medication use in the neonatal intensive care unit. Am. J. Perinatol..

[100] Rhone E.T., Carmody J.B., Swanson J.R., Charlton J.R. (2014). Nephrotoxic medication exposure in very low birth weight infants. J. Matern. Fetal Neonatal Med..

[101] Slater M.B., Gruneir A., Rochon P.A., Howard A.W., Koren G., Parshuram C.S. (2017). Identifying high-risk medications associated with acute kidney injury in critically ill patients: A pharmacoepidemiologic evaluation. Paediatr. Drugs.

[102] Hanna M.H., Askenazi D.J., Selewski D.T. (2016). Drug-induced acute kidney injury in neonates. Curr. Opin. Pediatr..

[103] Arcinue R., Kantak A., Elkhwad M. (2015). Acute kidney injury in ELBW infants (<750 grams) and its associated risk factors. J. Neonatal Perinat. Med..

[104] Fanos V., Cataldi L. (1999). Antibacterial-induced nephrotoxicity in the newborn. Drug Saf..

[105] Weitkamp J.H., Poets C.F., Sievers R., Musswessels E., Groneck P., Thomas P., Bartmann P. (1998). Candida infection in very low birth-weight infants: Outcome and nephrotoxicity of treatment with liposomal amphotericin B (AmBisome). Infection.

[106] Jeon G.W., Koo S.H., Lee J.H., Hwang J.H., Kim S.S., Lee E.K., Chang W., Chang Y.S., Park W.S. (2007). A comparison of AmBisome to amphotericin B for treatment of systemic candidiasis in very low birth weight infants. Yonsei Med. J..

[107] Goldstein S.L., Dahale D., Kirkendall E.S., Mottes T., Kaplan H., Muething S., Askenazi D.J., Henderson T., Dill L., Somers M.J. (2020). A prospective multi-center quality improvement initiative (NINJA) indicates a reduction in nephrotoxic acute kidney injury in hospitalized children. Kidney Int..

[108] Stoops C., Stone S., Evans E., Dill L., Henderson T., Griffin R., Goldstein S.L., Coghill C., Askenazi D.J. (2019). Baby NINJA (Nephrotoxic Injury Negated by Just-in-Time Action): Reduction of Nephrotoxic Medication-Associated Acute Kidney Injury in the Neonatal Intensive Care Unit. J. Pediatr..

[109] Ellison R.C., Pecham G.J., Lang P., Talner N.S., Lerer T.J., Lin L., Dooley K.J., Nadas A.S. (1983). Evaluation of the preterm infant for patent ductus arteriosus. Pediatrics.

[110] McCurnin D., Seidner S., Chang L.Y., Waleh N., Ikegami M., Petershack J., Yoder B., Giavedoni L., Albertine K.H., Dahl M.J. (2008). Ibuprofen-induced patent ductus arteriosus closure: Physiologic, histologic, and biochemical effects on the premature lung. Pediatrics.

[111] Ohlsson A., Shah P.S. (2018). Paracetamol (acetaminophen) for patent ductus arteriosus in preterm or low birth weight infants. Cochrane Database Syst. Rev..

[112] Kaempf J.W., Wu Y.X., Kaempf A.J., Kaempf A.M., Wang L., Grunkemeier G. (2012). What happens when the patent ductus arteriosus is treated less aggressively in very low birth weight infants?. J. Perinatol..

[113] Clyman R.I. (2013). The role of patent ductus arteriosus and its treatments in the development of bronchopulmonary dysplasia. Semin. Perinatol..

[114] Antonucci R., Fanos V. (2009). NSAIDs, prostaglandins and the neonatal kidney. J. Matern. Fetal Neonatal Med..

[115] Hirt D., Van Overmeire B., Treluyer J.M., Langhendries J.P., Marguglio A., Eisinger M.J., Schepens P., Urien S. (2008). An optimized ibuprofen dosing scheme for preterm neonates with patent ductus arteriosus, based on a population pharmacokinetic and pharmacodynamic study. Br. J. Clin. Pharmacol..

[116] Bakshi S., Koerner T., Knee A., Singh R., Vaidya R. (2020). Effect of fluid bolus on clinical outcomes in very low birth weight infants. J. Paediatr. Pharmacol. Ther..

[117] Thompson E.J., Zimmerman K.O., Gonzalez D., Foote H.P., Park S., Hill K.D., Hurst J.H., Hornik C.D., Chamberlain R.C., Gbadegesin R.A. (2024). Population Pharmacokinetics of Caffeine in Neonates with Congenital Heart Disease and Associations with Acute Kidney Injury. J. Clin. Pharmacol..

[118] Puzanov A., Tkachuk V., Maksymenko A. (2023). Acute kidney injury after arterial switch operation: Incidence, risk factors, clinical impact—A retrospective single-center study. Ren. Fail..

[119] Harer M.W., Pope C.F., Conaway M.R., Charlton J.R. (2017). Follow-up of acute kidney injury in neonates during childhood years (FANCY): A prospective cohort study. Pediatr. Nephrol..

[120] Wong J.H., Selewski D.T., Yu S., Leopold K.E., Roberts K.H.B., Donohue J.E., Ohye R.G., Charpie J.R., Goldberg C.S., DeWitt A.G. (2016). Severe acute kidney injury following stage 1 norwood palliation: Effect on outcomes and risk of severe acute kidney injury at subsequent surgical stages. Pediatr. Crit. Care Med..

[121] Adegboyega O.O., Singh Y., Bhutada A., Kupferman J.C., Rastogi S. (2022). Recurrent acute kidney injury in preterm neonates is common and associated with worse outcomes and higher mortality. Pediatr. Res..

[122] Cleper R., Shavit I., Blumenthal D., Reisman L., Pomeranz G., Haham A., Friedman S., Goldiner I., Mandel D. (2019). Neonatal acute kidney injury: Recording rate, course, and outcome: One center experience. J. Matern. Fetal Neonatal Med..

[123] Vincent K., Rutledge A., Laney Z., Newman J.C., Selewski D.T., Steflik H.J. (2023). Recurrent neonatal acute kidney injury: Incidence, predictors, and outcomes in the neonatal intensive care unit. J. Perinatol..

[124] Rutledge A.D., Griffin R.L., Vincent K., Askenazi D.J., Segar J.L., Kupferman J.C., Rastogi S., Selewski D.T., Steflik H.J. (2024). Incidence, Risk Factors, and Outcomes Associated with Recurrent Neonatal Acute Kidney Injury in the AWAKEN Study. JAMA.

[125] Greenberg J.H., Coca S., Parikh C.R. (2014). Long-term risk of chronic kidney disease and mortality in children after acute kidney injury: A systematic review. BMC Nephrol..

[126] Lebel A., Teoh C.W., Zappitelli M. (2020). Long-term complications of acute kidney injury in children. Curr. Opin. Pediatr..

[127] Chawla L.S., Eggers P.W., Star R.A., Kimmel P.L. (2014). Acute kidney injury and chronic kidney disease as interconnected syndromes. N. Engl. J. Med..

[128] Chimenz R., Chirico V., Cuppari C., Ceravolo G., Concolino D., Monardo P., Lacquaniti A. (2022). Fabry disease and kidney involvement: Starting from childhood to understand the future. Pediatr. Nephrol..

[129] Mammen C., Al Abbas A., Skippen P., Nadel H., Levine D., Collet J., Matsell D.G. (2012). Long-term risk of CKD in children surviving episodes of acute kidney injury in the intensive care unit: A prospective cohort study. Am. J. Kidney Dis..

[130] Menon S., Kirkendall E.S., Nguyen H., Goldstein S.L. (2014). Acute kidney injury associated with high nephrotoxic medication exposure leads to chronic kidney disease after 6 months. J. Pediatr..

[131] Harer M.W., Charlton J.R., Tipple T.E., Reidy K.J. (2020). Preterm birth and neonatal acute kidney injury: Implications on adolescent and adult outcomes. J. Perinatol..

[132] Keijzer-Veen M.G., Schrevel M., Finken M.J., Dekker F.W., Nauta J., Hille E.T., Frölich M., van der Heijden B.J., Dutch POPS-19 Collaborative Study Group (2005). Microalbuminuria and lower glomerular filtration rate at young adult age in subjects born very premature and after intrauterine growth retardation. J. Am. Soc. Nephrol..

[133] Cheung Y.F., Wong K.Y., Lam B.C.C., Tsoi N.S. (2004). Relation of arterial stiffness with gestational age and birth weight. Arch. Dis. Child..

[134] Franke D., Völker S., Haase S., Pavicic L., Querfeld U., Ehrich J.H., Zivicnjak M. (2010). Prematurity, small for gestational age and perinatal parameters in children with congenital, hereditary and acquired chronic kidney disease. Nephrol. Dial. Transplant..

[135] Bacchetta J., Harambat J., Guy B., Putet G., Cochat P., Dubourg L. (2009). Long term renal outcome of children born preterm: A regular follow-up is needed. Arch. Pediatr..

